# Efficacy and Safety of Weekly Ultraviolet Germicidal Irradiation for the Reuse of N95 Filtering Respirators

**DOI:** 10.7759/cureus.18233

**Published:** 2021-09-24

**Authors:** Keitaro Nakamoto, Takeshi Saraya, Narishige Ishikawa, Sunao Mikura, Yuki Yoshida, Naokatsu Fukukawa, Takako Taneoka, Teppei Shimasaki, Daisuke Kurai, Haruyuki Ishii

**Affiliations:** 1 Respiratory Medicine, Kyorin University School of Medicine, Mitaka, JPN; 2 Department of Infection Control, Kyorin University School of Medicine, Mitaka, JPN; 3 Infectious Disease, Kyorin University School of Medicine, Mitaka, JPN; 4 Department of Respiratory Medicine, Kyorin University School of Medicine, Mitaka, JPN

**Keywords:** ultraviolet germicidal irradiation, reuse, n95 filtering facepiece respirators, fit test, covid-19

## Abstract

Background

During the ongoing coronavirus disease (COVID-19) pandemic, N95 filtering facepiece respirators (N95 respirators) are in short supply in many countries. Considering this, the Centers for Disease Control and Prevention suggested reusing N95 respirators and recommended the use of ultraviolet germicidal irradiation (UVGI) for sterilizing the respirators. However, only a few reports have described UVGI protocols for sterilizing the N95 respirators for reuse. Therefore, in this study, we aimed to develop and evaluate a novel method for the reuse of N95 respirators after sterilization by UVGI.

Methods

Before conducting the study, the function of N95 respirators after multiple UVGI with a total dose of up to 10 J (1 J/cm^2^ or more per dose) was assessed by measuring the particle collection efficiency and ventilation resistance. The participants used N95 respirators during work if they passed the fit test. After use, the respirators were sterilized using UVGI (1 J/cm^2^) and stored in a breathable paper bag for a week. The procedure was repeated up to three times after confirming the successful results of the fit tests.

Results

The particle collection efficiency without UVGI was 96.7%, while those after one, five, and 10 cycles of UGVI were 96.8%, 97.2%, and 97.2%, respectively. Ventilation resistance without UVGI was 42 Pa, and 43 Pa, 42 Pa, and 41 Pa after one, five, and 10 cycles of UVGI, respectively, which satisfied the Japanese national certification standard DS2. All 43 participants passed the fit test before the first reuse, and 39 participants (90.7%) completed the entire study protocol. The results of this study showed that N95 respirators could be used safely after repeated UVGI treatment.

Conclusions

This study developed a novel method for reusing the N95 respirators. A few cycles of UV radiation N95 masks retain their functionalities and can be reused with proper UVGI.

## Introduction

Appropriate use of personal protective equipment (PPE) including N95 filtering facepiece respirators (N95 respirators) protects healthcare workers from the severe acute respiratory syndrome coronavirus 2 (SARS-CoV-2) [1]. During the ongoing coronavirus disease (COVID-19) pandemic, N95 respirators are in short supply in many countries due to a dramatic increase in demand. Considering this, the Centers for Disease Control and Prevention have suggested reusing the N95 respirators [2]. Although novel treatment and the availability of vaccines against COVID-19 will improve this situation, the waves of resurgence have not subsided worldwide. Hence, ensuring a continuous supply of PPE globally is challenging.

Recently, Millis et al. reported that N95 respirators can be sterilized and reused after appropriate ultraviolet germicidal irradiation (UVGI) [3]. Previous reports have also shown that UV irradiation of ≥ 1 J/cm^2^ inactivates various viruses, including the avian influenza A virus (H5N1), on N95 respirators [4, 5]. However, few reports have verified whether N95 respirators can be sterilized by UVGI and reused. Most N95 respirators described in the literature are not widely distributed in Japan. Along with a shortage of N95 respirators, the asymptomatic carrier state of the SARS-CoV-2 infection has led to COVID-19 clusters in community hospitals and/or health care institutions worldwide, leading to ongoing critical concerns. Hence, developing a more accurate and safer method for reusing N95 respirators is urgently required. Therefore, we conducted this study wherein we verified whether multiple UVGI treatments could sterilize N95 respirators for reuse.

## Materials and methods

Prior to enrolling the patients, the N95 respirator’s performance was evaluated in view of collection efficiency and ventilation resistance after UVGI irradiation with various doses.

The way of UVGI and the method of N95 respirator function after UVGI

Ultraviolet Germicidal Irradiation

**Figure 1:**
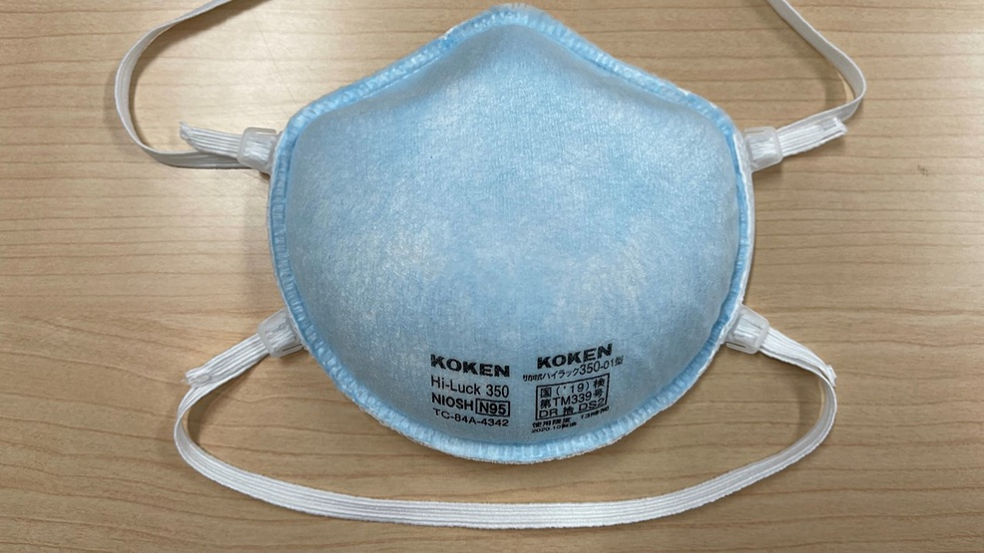
The Hi-Luck 350 N95 respirator The N95 filtering facepiece respirator, Hi-Luck 350 (Koken Ltd., Tokyo, Japan) used in this study.

We used UVDI-360 Room Sanitizer (UVDI, Valencia, CA, USA) as an ultraviolet germicidal irradiation system to sterilize used N95 respirators. This device had four 254 nm UV-C lamps arranged in a parallel configuration. A cup-shaped N95 respirator, the Hi-Luck 350 (Koken Ltd., Tokyo, Japan) (Figure 1), was used in this study.

**Figure 2:**
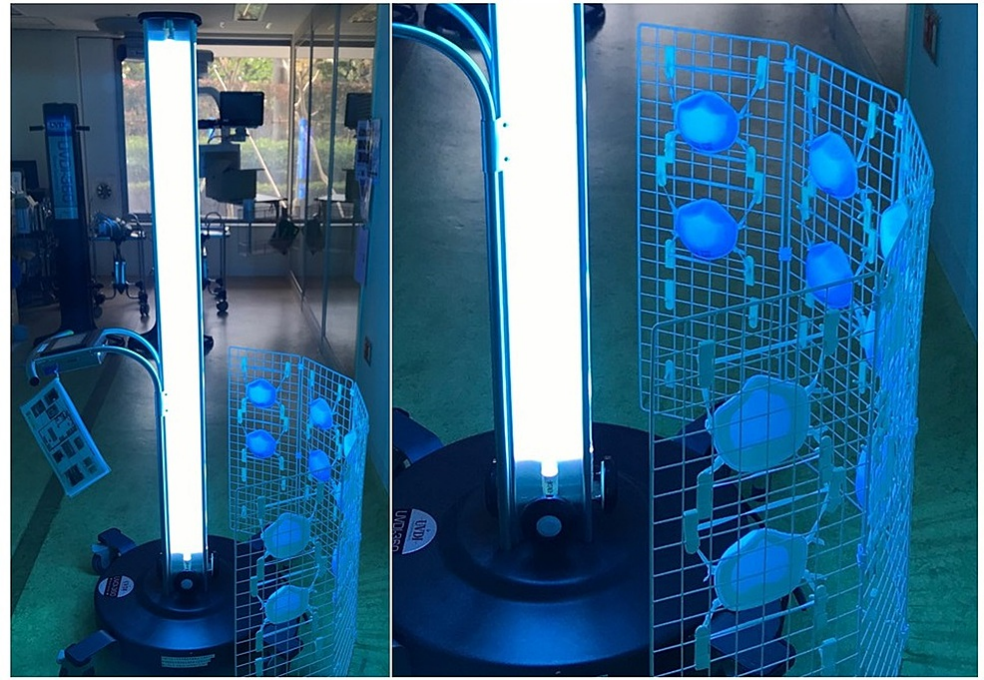
Ultraviolet germicidal irradiation This shows the process of ultraviolet germicidal irradiation on the N95 filtering facepiece respirators (N95 respirators) in this study. Using this method, the N95 respirators are positioned on a metal fence, at a distance of 50 cm from the ultraviolet germicidal irradiation system, and irradiated on both the front and back surfaces, for 10 minutes (≧ 1 J/cm^2^ per one side) per surface.

N95 respirators were placed at a distance of 50 cm from the UVGI system and irradiated on both sides for 10 min (Figure 2). This process was conducted as one cycle, which corresponded to a UV-C of 1 J/cm^2^ or more. The total amount of UV-C irradiance energy (J/cm^2^) was measured using a radiometer, UVC-254SD ST (SATOTECH, Kanagawa, Japan).

Evaluation of the N95 respirator function after UVGI

First, we evaluated the performance of the N95 respirators using an automated filter tester 8130 (TSI, Shoreview, MN, USA). The collection efficiency was measured using NaCl particles at a test airflow rate of 85 L/min (pass criteria ≥ 95%). Ventilation resistance was measured at a test airflow rate of 40 L/min (pass criteria ≤ 50 Pa). These conditions are defined by the Japanese national certification standard DS2, which is considered a standard certificate of performance comparable with that of the National Institute for Occupational Safety and Health (NIOSH) [6]. In this study, the N95 respirator’s performance was measured after UVGI irradiation with 0, one, five, and 10 cycles (0, ≥ 1, ≥ 5, ≥ 10 J/cm^2^, respectively). Each condition was evaluated using three N95 respirators.

Reuse of UVGI N95 Respirator to the participants

Study Participants

After evaluating the N95 respirator performance, we conducted this study from December 2020 to March 2021 at Kyorin University Hospital. We enrolled healthcare workers (doctors or nurses) who used N95 respirators at work. All participants worked in the endoscopy room, outpatient, or inpatient wards. Most N95 respirators were used by healthcare workers when collecting respiratory samples or during bronchoscopies. In this study, only the cup-shaped N95 respirator, namely, the Hi-Luck 350 was used.

N95 Respirator Reuse Protocol

**Figure 3:**
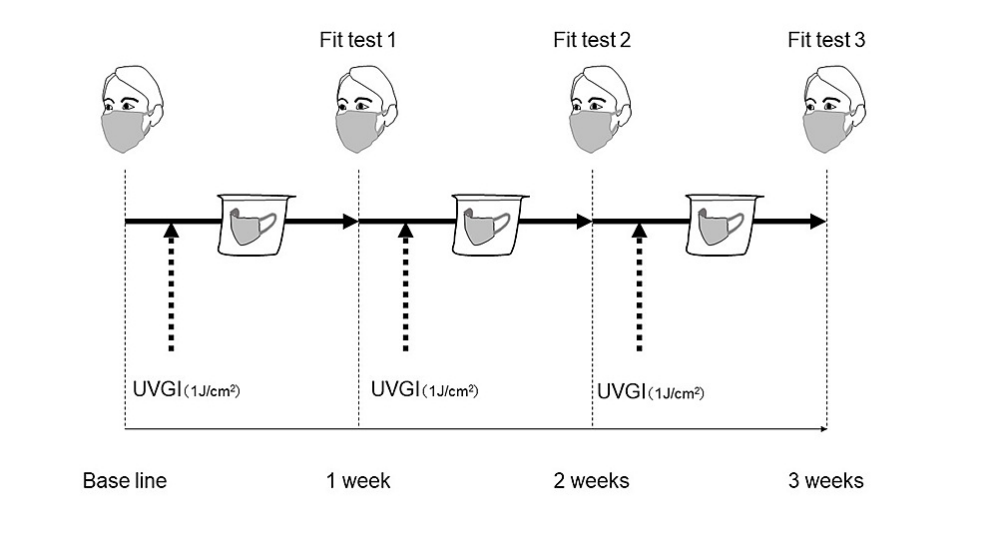
Schematic of the study protocol Prior to enrolling the patients, the N95 respirator’s performance was guaranteed in the view of collection efficiency and ventilation resistance after UVGI irradiation with 0,1,5, and 10 cycles (1J/cm^2^ per procedure both inner and outer surface of the N95 respirator's). The participants performed a baseline fit test before using an N95 respirator. The N95 respirators were stored for one week without being used and were allowed to be reused a second time if the participants passed the fit test. These procedures were repeated for up to three weeks. Participants who could not pass the fit test (fit test one) were eliminated from the subsequent steps of the study. Ultraviolet germicidal irradiation (UVGI) was performed on both the front and back surfaces, for 10 minutes (≧ 1 J/cm^2^ per one side) per surface.

The reuse protocol for N95 respirators is illustrated in Figure 3 based on the method described in our previous report [7]. Briefly, participants performed a baseline fit test before using an N95 respirator. The fit test was quantitatively evaluated using the MT-03 mask fitness tester (Sibata Scientific Technology Ltd., Saitama, Japan) [7]. This device measures the number of particles on the internal and external surfaces of the respirators. The threshold for allowable air leakage rate was defined as less than 5% [7].

All participants were required to pass the fit test before using an N95 respirator. If participants could not pass the fit test first, they were excluded from the study. The used N95 respirators during a work shift were sterilized by UVGI at ≥ 1 J/cm2 and stored in a breathable paper bag. Subsequently, the N95 respirators were stored for one week without being used because of the survival time of SARS-CoV-2 on the surfaces [8]. The N95 respirators were allowed to be reused a second time if the participants passed the fit test. These procedures were repeated for up to three weeks. Participants who could not pass the fit test (fit test one) were eliminated from the subsequent steps of the study.

We recorded age, years of work experience, the reuse duration of N95 respirators per shift, and fit test pass rate. The primary outcome was the pass rate of successful reuse over three observational weeks through the four-fold fit tests.

Statistical analysis

Data are presented as the median and interquartile range (IQR) for continuous variables and as the number (percentage) for categorical variables.

Differences between the groups were assessed using the Mann-Whitney U test for continuous variables and the Fisher’s exact test for categorical variables. Statistical significance was set at P <0.05. All statistical analyses were performed using IIBM Corp. Released 2019. IBM SPSS Statistics for Windows, Version 26.0. Armonk, NY: IBM Corp. Informed consent was obtained from all participants. This study was approved by the Institutional Review Board of Kyorin University (approval number: 1697) and performed according to the Declaration of Helsinki.

## Results

N95 respirator function after UVGI

Table 1 shows the function of the N95 respirator after UVGI treatment. The particle collection efficiency without UVGI was 96.7% (IQR: 96.4-96.7%), while those after one, five, and 10 cycles of UGVI were 96.8% (IQR: 96.2-96.1%), 97.2% (96.2-97.4%), and 97.2% (96.2-97.4%), respectively. Ventilation resistance without UVGI was 42 Pa (IQR: 41-42 Pa), and 43 Pa (IQR: 42-43 Pa), 42 Pa (IQR: 41-44 Pa), and 41 Pa (IQR: 41-42 Pa) after one, five, and 10 cycles of UVGI, respectively. These results satisfied the Japanese national certification standard DS2, which is considered equivalent to the NIOSH standard certification for N95 respirators [6].

**Table 1:** Performance of the N95 filtering facepiece respirators after ultraviolet germicidal irradiation Data are presented as the median. IQR: interquartile range

Irradiation cycle (Times)	Particle collection efficiency (%)	Ventilation resistance (Pa)
0	96.7 (96.4-96.7)	42 (41-42)
1	96.8 (96.6-97.1)	43 (42-43)
5	97.2 (96.5-97.4)	42 (41-44)
10	96.7 (96.6-96.9)	41 (41-42)

Patient characteristics and the protocol results

We successfully enrolled 48 participants, but three participants did not pass the baseline fit test, and two participants withdrew from the study. A total of 43 participants, comprising 18 doctors and 25 nurses were enrolled (Table 2). Of these, 30 participants (69.8%) were women, with a median age of 33 years (IQR: 29-38 years). The median time of reuse of the N95 respirator per shift was 2.3 hr (IQR: 1.9-2.5 hr). All 43 participants successfully passed fit test one. However, only 39 (90.7%) participants completed the protocol because four (9.3%) participants were eliminated during fit test two (n=2) and fit test three (n=2).

**Table 2:** Participant characteristics

	Total (N=43)	Pass (N=39)	Failure (N=4)	P value
Sex				
Women	30 (69.8)	26 (66.7)	4 (100.0)	0.297
Men	13 (30.2)	13 (33.3)	0 (0.0)	
Age	33 (29-38)	33 (30-38)	27 (25-36)	0.181
Years of work experience	8 (6-12)	8 (6-12)	6 (4-14)	0.319
Type of healthcare worker				
Doctor	18 (41.9)	18 (46.2)	0 (0.0)	0.127
Nurse	25 (58.1)	21 (53.8)	4 (100.0)	
Mask-use hours/ One-shift use	2.3 (1.9-2.5)	2.3 (1.8-2.5)	2.4 (2.3-2.6)	0.280
Data are presented as the median (interquartile range; IQR) or number (%).				

However, no significant differences were noted regarding sex ratio, age, years of work experience, type of occupation, and reuse duration of the N95 respirator per shift. Throughout the study period, no participants suffered from SARS-CoV-2 infection.

## Discussion

This study confirmed that repeated UVGI at more than 1 J/cm^2^ per cycle up to 10 J/cm^2^ was tolerable to reuse in the aspect of the function of N95 respirators as examined by the ventilation resistance. A total of 90.7% of the participants successfully completed the protocol, passing three fit tests.

The N95 respirator can capture more than 95% of 0.3 μm airborne particles [9], which is imperative for medical staff who are exposed to patients with infectious diseases, such as pulmonary tuberculosis, measles, chickenpox, and SARS-CoV-2. As such, the COVID-19 pandemic in 2020 caused a worldwide shortage of N95 respirators. To date, there is limited evidence on the safety and efficacy of reusing N95 respirators.

A study by Degesys et al. reported that 38.2% of the 68 participants who reused N95 respirators failed the fit test [10]. Interestingly, the failure rate differed according to the type of N95 respirator used, and the duckbill-shaped N95 respirators had a higher failure rate than those that were dome-shaped.

In our previous study, we reported that N95 respirators are durable and can be reused a total of four times at weekly intervals [7]. However, the risk of exposure to live SARS-CoV-2 on the surfaces of N95 respirators was eliminated only after one-week intervals for every reuse.

On the other hand, the use of UVGI and hydrogen peroxide (H_2_O_2_) to inactivate SARS-CoV-2 for the reuse of N95 respirators has been reported. UVGI is a sterilization method using UV-C and is effective against influenza viruses and bacteria with spores [4]. Recently, some reports have applied UV-C irradiation for inactivating SARS-CoV-2 [11, 12]. Treatment with H_2_O_2_ can also inactivate viruses and highly resistant bacterial spores [13, 14]. However, these methods cannot be used if the N95 respirators contain cellulose or cellulose-based materials [15]. A room with well-controlled airflow is also necessary. For these reasons, our study used UVGI to sterilize N95 respirators. Furthermore, we assessed the effectiveness of the N95 respirators before conducting the study by sterilizing them using UVGI at up to 10 cycles per 1 J/cm^2^. Therefore, we allowed the N95 respirators to rest for a certain period before conducting this research.

This study showed a high success rate of reusing N95 respirators that were sterilized by UVGI at weekly intervals. We demonstrated that our protocol is a safer and more reliable method for reusing N95 respirators among healthcare workers.

This study had several limitations. First, this was a single-center study. In addition, the reused N95 respirators were sterilized using UVGI every week, rather than daily. Furthermore, we examined only cup-shaped N95 respirators as they had shown optimal results in our previous study [7]. Secondly, we did not perform the viral culture of SARS-CoV-2 from which surface of N95 respirator, but sufficient weekly UVGI for eliminating SARS-CoV-2 with time decontamination (one-week interval for reuse). Further research investigating various types of N95 respirators and/or institution-based UVGI protocols with the viral culture is warranted.

## Conclusions

In conclusion, this study shows that the particle collection efficiency and ventilator resistance of cup-shaped type N95 respirators did not change after multiple cycles of UVGI. Based on the results of the quantitative fit tests, these N95 respirators are safe and effective for reuse. Thus, this study outlines a novel method for achieving sterilization of the N95 respirator in a short duration and prolonging its use.
